# Determinants and Mitigating Factors of Brain Drain among Ghanaian Nurses: Insights from Nurse Managers in Northern Ghana—A Qualitative Inquiry

**DOI:** 10.1155/2024/8862991

**Published:** 2024-08-17

**Authors:** Mudasir Mohammed Ibrahim, Abubakari Wuni, Waliu Jawula Salisu, Abdul-Malik Abdulai, Theresah Owusua, Brenda Abena Nyarko, Abdul-Malik Sayibu, Hannah Buasilenu, Abdulai Issahaka Baako, Iddrisu Sisala Mohammed

**Affiliations:** ^1^ Nurses' and Midwives' Training College, Tamale, Ghana; ^2^ College of Nursing University of Kentucky, Lexington, KY, USA; ^3^ Cambridge University Hospitals NHS Foundation Trust, Cambridge, UK; ^4^ Catholic University of Ghana, Sunyani, Ghana; ^5^ Elaine Marieb College of Nursing University of Massachusetts, Amherst, MA, USA; ^6^ Department of Child Health Hopexchange Medical Center, Kumasi, Ghana; ^7^ Sang Health Center Ghana Health Service, Accra, Ghana

## Abstract

**Background:**

Nurse emigration, often termed “brain drain,” poses significant challenges to Ghana's healthcare sector.

**Aim:**

This study explores nurse managers' perspectives on determinants and strategies for mitigating nurse emigration in Northern Ghana.

**Methods:**

Sixteen nurse managers were interviewed using semistructured interviews between October and December 2023. Purposive sampling was used to select the participants. QDA Miner Lite version 6 was used for systematic coding and thematic data analysis, following the conventional content analysis approach.

**Results:**

Following data encoding and classification, the study identified three primary categories: determinants of brain drain, impact of brain drain on the healthcare system, and mitigating factors of brain drain.

**Conclusion:**

The study reveals that various factors, including inadequate pay, limited opportunities for career growth, and lack of access to technology, drive the brain drain among Ghanaian nurses. These lead to negative impacts on the healthcare system, such as increased workload, reduced patient satisfaction, and a shortage of skilled nurses. To tackle this issue, solutions including offering career advancement opportunities and improving salaries and working conditions among others have been highlighted to mitigate the brain drain among Ghanaian nurses.

## 1. Introduction

The migration of nurses, particularly from low- and middle-income countries to those with higher incomes, is a major global issue that has gained worldwide attention. Unfortunately, the recruitment tactics used by many of these low- and middle-income countries, especially in Africa, have made the situation worse as they face difficulties retaining their nursing graduates who their educational institutions have trained [1].

This outflow of skilled professionals from developing countries to developed countries is commonly referred to as the “brain drain” [2]. The term was first used by the British Royal Society in the 1960s to describe the large departure of professional scientists, physicians, and university lecturers from developing countries. It has since become a major concern globally [3, 4]. According to a 2015 report by the World Health Organization, developed countries are experiencing a severe shortage of healthcare workforce, despite dealing with an aging population and an increased prevalence of chronic illnesses. This shortage of highly skilled nurses is not only due to needs-based factors but also because of the high demand for nurses, which is expected to increase in the coming years. Consequently, it is highly likely that this high demand will severely deplete the pool of skilled nurses in developing countries [5].

Nurse migration has been observed across most Organization for Economic Co-operation and Development (OECD) countries, with a significant increase in the number of nurses migrating to these countries since the early 2000s. In fact, the numbers have more than doubled between 2000 and 2016. Additionally, between 2011 and 2018, the figures increased by about 20%, constituting over 7% of the nursing workforce in the OECD region. In 2021 alone, OECD countries employed an overwhelming 550,000 nurses [6, 7], indicating the need for constructive measures to manage this phenomenon effectively.

Ghana's history of migration is characterized by dynamic and multifaceted factors, deeply rooted in historical events and a tradition of population mobility. Urban migration, in particular, has played a pivotal role in livelihood strategies within the country. In the context of professional movement, nurses often transition across various levels of healthcare facilities, from rural to urban settings, and from clinical and research roles to administrative positions [8, 9].

Recent policy shifts in Ghanaian healthcare, such as initiatives aimed at improving salaries, enhancing working conditions, and providing career advancement opportunities, have attempted to mitigate nurse emigration [10, 11]. However, the effectiveness of these policies in addressing the root causes of nurse migration remains a critical concern [3, 12].

Despite significant advancements in the Ghanaian healthcare system over the past three decades [13], the country grapples with a substantial loss of healthcare professionals to foreign countries, presenting a critical challenge [14].

A considerable number of Ghanaian nurses choose to migrate to developed countries such as the United Kingdom, the United States, Australia, Canada, and more recently, Barbados and the United Arab Emirates, in pursuit of better job opportunities [15]. According to the Ghana Registered Nurses' and Midwives' Association, over 6,000 Ghanaian nurses have relocated to foreign countries within a two-year period, with an additional 14,000 nurses seeking financial clearance for the same purpose [16]. This trend is further underscored by data from the United Kingdom's National Health Service (NHS), which recorded a notable increase in the number of healthcare workers, including nurses, with Ghanaian heritage [10]. According to Iddrisu [17], about 60% of Ghanaian nurses harbor intentions to leave the country and seek employment opportunities elsewhere.

The decision of nurses to migrate is influenced by a complex interplay of push and pull factors. Pull factors pertain to attractive conditions present in the health systems of destination countries, while push factors refer to undesirable features of the healthcare systems in origin countries [18]. In Ghana, a range of push factors, including low salaries, limited access to modern technology, restricted educational opportunities, low job satisfaction, and political instability, contribute to nurses' motivation to seek opportunities abroad [3, 18, 19]. Failure to address this trend could lead to increased clinical workloads, decreased quality of care, and heightened mortality and morbidity rates due to reduced access to essential healthcare services [5, 20]. Given the urgency of this issue, this qualitative study was conducted to explore the perspectives of nurse managers regarding the determinants and mitigating factors of nurse migration, specifically focusing on Northern Ghana.

## 2. Methods

This qualitative study used a conventional content analysis approach to delve into the contributing and mitigating factors of nurse migration in Ghana. Content analysis was deemed appropriate as it is suited for describing phenomena with limited understanding or fragmented knowledge about them [21, 22]. Given the ambiguous nature of the phenomenon of nurse brain drain in Northern Ghana, content analysis was chosen to bring clarity and insight into this complex issue.

Participating nurse managers were purposefully selected from three hospitals in Tamale (Tamale Teaching Hospital, Seventh Day Adventist, and Tamale West Hospital), the capital city of the Northern Region. Participants were purposively selected from different departments within each hospital. The study included 16 participants, 9 females and 7 males, all with a minimum of 5 years of managerial experience. The sample size of sixteen was determined based on the principle of data saturation, where we observed that no new insights were emerging from additional interviews, ensuring that the sample size was sufficient to capture the breadth and depth of perspectives on the topic. The criteria for selecting participants included factors such as years of managerial experience (minimum 5 years), current active service in clinical or managerial roles, and willingness or consent to participate. The study relied on nurse managers for their frontline leadership roles and extensive workforce experience, aiming to comprehensively understand and address the issue of nurse attrition in the region.

Data were collected through in-depth face-to-face interviews that lasted 45 to 60 minutes. The interviews were conducted in quiet settings in participants' offices, as they mostly preferred.

The interview process commenced with questions aligned with the study's objectives, followed by probing inquiries to delve deeper into specific aspects. Each interview began with an open-ended question, such as, “As a nurse manager, could you share your insights on the issue of brain drain among nurses?” This approach fostered comprehensive discussions and elicited rich qualitative data. Depending on the responses, further probing questions were asked. The data collection continued until data saturation was reached, ensuring comprehensive coverage of relevant insights.

The data analysis employed a conventional approach without predetermined categorization structures. This method unfolded over three phases: preparation, organization, and report writing. Initially, in the preparation phase, each interview served as a unit of analysis. Transcripts were meticulously transcribed verbatim and iteratively reviewed to grasp overarching categories.

All audio recordings were transcribed verbatim by two independent transcribers to ensure accuracy. The transcripts were then imported into a qualitative data analysis software, QDA Miner Lite version 6, for systematic coding and thematic analysis. To begin the analysis, the researchers engaged in open coding, wherein initial concepts and patterns were identified. Axial coding was employed to categorize and relate codes to each other, allowing for the emergence of preliminary themes.

During the organization phase, units of meaning within each interview were identified, condensed, and openly coded. Similar codes were then grouped into subcategories and overarching main categories. Finally, in the reporting phase, the latent meaning of the data was presented as the study's results [23].

Efforts were made to ensure the reliability and conformability of the findings. The credibility of the findings was further bolstered by meticulous attention to the content analysis process, including selecting meaningful units, categorizing data, and recognizing similarities and differences among categories. To this end, ample time was dedicated to data collection and analysis. A member check was also conducted (Graneheim & Lundman, 2004), and two other authors independently reviewed the data for peer validation.

The team obtained ethical clearance from the Tamale Teaching Hospital ethical unit with reference number TTH/R&D/SR/267. Before the interviews, participants received detailed information about the study, including its objectives, potential risks, and benefits. Each participant provided informed consent, ensuring their voluntary and confidential participation in the study.

## 3. Results

**Figure 1:**
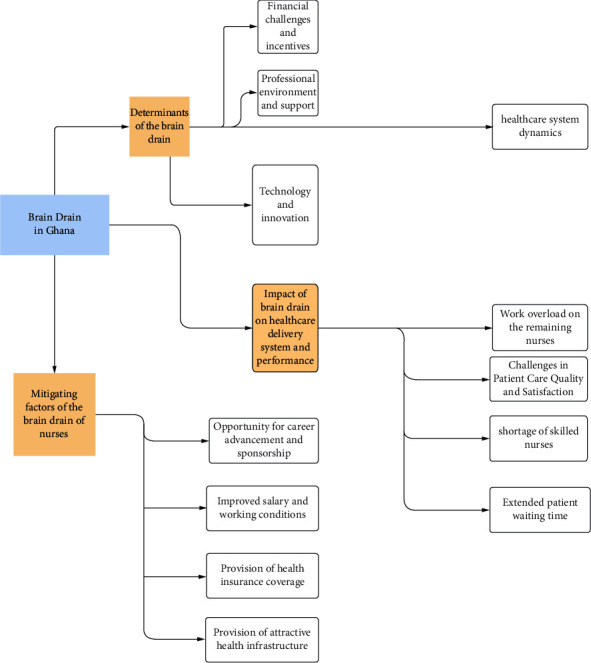
Categories and subcategories that emerged from the interview data.

In this study, a diverse group of participants contributed valuable insights, enabling the authors to draw meaningful conclusions regarding the brain drain phenomenon in Ghana.  Table 1 presents the demographic data of the participants. The analysis of the findings highlighted three major categories along with 12 subcategories, as seen in Figure 1.

### 3.1. Determinants of the Brain Drain

The determinants of brain drain have emerged as one of the main categories, highlighting the factors that drive the phenomenon of brain drain. Understanding these determinants is crucial for policymakers and researchers aiming to address and mitigate the consequences of brain drain. It explores the complex interactions between factors influencing the brain drain issue in Ghana, focusing on four subcategories: Financial Challenges and Incentives, Professional Environment and Support, Healthcare System and Dynamics, and Technology and Innovation.

#### 3.1.1. Financial Challenges and Incentives

All the participants in this study revealed that one of the main reasons for nurses leaving the country is the financial challenges and incentives. Nurses' earnings are inadequate for significant expenses such as buying a car or a house. To achieve such goals, nurses often have to take high-interest loans, which causes financial strain. As a result, in retirement, nurses often face difficult living conditions. On the other hand, it was noted that nurses who have migrated to foreign countries have better economic prospects and can often achieve their goals. They can enjoy a better quality of life and often return to their home country to contribute significantly. Also, some participants mentioned delayed promotions, the search for a better life for themselves and family, and the absence of health insurance coverage as the reasons why nurses travel out of the country.

*“I think our salary is just from hand to mouth. Before you can do any big project, you have to go for a loan. Before you get a house or car, you have to go for a loan and if you look at those nurses who have retired, their living conditions are not that appreciable. I have seen a colleague who has gone outside for less than two years and things have changed. He is able to get a house and he has his own car. ‘Why won't you also want to go and get those things?'”* (Participant 5).

*“They will send the money home so that they will take good care of the family they have left”* (Participant 3).

*“There is no proper health insurance for workers who work in hospitals. When you are sick, you still have to take care of yourself and pay out of pocket”* (Participant 14).

#### 3.1.2. Professional Environment and Support

Our findings showed that nurses are undervalued and disrespected in the healthcare sector, a significant factor driving their emigration. The difference in status between medical doctors and nurses is one reason for this, as several participants pointed out. They emphasised that even when a nurse has advanced degrees and extensive experience exceeding that of a newly qualified medical doctor, healthcare facilities often fail to acknowledge their expertise, and the nurse is unable to attain a position of authority or denied a promotion to merit their experience. This is in contrast to doctors who are frequently promoted to higher ranks. It is high time that healthcare organizations recognize the qualifications and expertise of nurses and treat them with the respect they deserve. During the interviews, several participants expressed that the government policy mandates that a nurse who serves for a certain number of years should be given a study leave to pursue further programs if they wish to. Participants noted that workplace bullying and discrimination against nurses were prevalent in most health facilities, contributing to an unpleasant work environment.

*“When a nurse goes to school to acquire higher degrees and comes back, the highest rank they can attain is a chief nursing officer in the institution. They cannot go any higher”* (Participant 3).

*“You go to a district hospital where there is a medical superintendent. The medical superintendent has completed medical school, done his housemanship for just two years and comes out as a medical doctor. Whereas there is a nurse, who has been working there for more than 20 years and has experience sometimes to the extent that some have higher degrees than the medical doctor. They still become subservient. The only way the government can stop nurses from going outside the country is when they start to see nurses as important and pivotal in the health delivery”* (Participant 9).

*“Then also, there are a lot of bureaucratic systems in place in our society. So, the government says that when you work a certain number of years, you should be allowed to go and get upgraded. You should go to school […] People have worked ten years, and they are still chasing study leave”* (Participant 6).

*…“So, for me, the main factor is bullying in the system and among us as nurses. It might come out subtly as if it is not there, but it's a big issue”* (Participant 11). *“[…] I see nurses discriminated against, and so they leave the shores of the country. For now, I don't see any good policy for nurses, which explains why people are leaving”* (Participant 15).

#### 3.1.3. Healthcare System Policies and Dynamics

Participants voiced concerns about the inadequate availability of essential logistics required for life-saving procedures. Furthermore, despite acquiring additional training in specialised skills, some nurses continue to face obstacles in accessing the necessary equipment. These collective challenges demotivate them, fueling a growing inclination to explore practice opportunities in other countries. The participants also cited their organization's lack of interaction with employees to address issues, the government's recent policy to export nurses, and the delay in recruitment of graduate nurses as significant factors propelling nurses to leave the country. Participants emphasised the importance of feeling respected and recognized in their professional endeavours, which does seem to exist.

*“You need consumables (such as gloves, cannulas, and syringes), but they are not there. When the patient comes in an emergency state, you have to write for a relative to go and buy it before you can attend to the person. You look at the patient as helpless but can't do anything. Common oxygen flow meters, you won't get them. You go, and then they ask you questions such as, ‘What are you using it for?' It's frustrating”* (Participant 12).

*“Even the government encourages the exodus of nurses; they started thinking they had enough and have been exporting to Barbados. So now, people have gone to Barbados, and they have realised that it is better there, so they are unwilling to return”* (Participant 9).

#### 3.1.4. Technology and Innovation

According to our findings, one of the main reasons why nurses are leaving their home countries is their curiosity about modern technology. Many nurses are highly motivated to practice in other countries after seeing the technological advancements on television and social media.

Although they may rely on manual methods in their home countries, they aspire to experience the technologically advanced lifestyle available in foreign countries. This curiosity becomes a compelling driving force for them.

*“We see it on TV and on social media that this time, this is the current trend that is going on, and we are still doing the old-fashioned things. Sometimes, out of curiosity, you will want to go and taste life on the other side of the island”* (Participant 2).

### 3.2. Impact of Brain Drain on Healthcare Delivery System and Performance

One of the major categories was the impact of brain drain on the healthcare delivery system and performance. The findings unveil the multifaceted repercussions of brain drain on healthcare provision, shedding light on healthcare systems' complexities. The subcategories included the Shortage of Skilled Nurses, Challenges of Patient Care Quality and Satisfaction, Work Overload on Remaining Nurses, and Extended Patient Waiting Time.

#### 3.2.1. Shortage of Skilled Nurses

Our findings indicate that nurses with significant expertise and experience are predominantly those leaving the country. Participants underscored the substantial loss incurred by the departure of these experienced nurses, emphasising the critical benefits of retaining them, mainly as patient care demands have heightened in recent years. Furthermore, they highlighted the potential risks to the country's ability to meet its targets for the Sustainable Development Goals (SDGs) if the shortage of skilled nurses within the healthcare system persists. The ongoing exodus of proficient nurses could severely undermine the healthcare system's capacity to deliver quality care, impeding progress towards achieving the SDGs. This scenario necessitates urgent attention and strategic interventions from policymakers to mitigate the adverse impacts and ensure a stable and competent nursing workforce.

*“…It is affecting the nursing profession in Ghana; those who have the experience and knowledge in nursing are rather leaving. Meanwhile, they have been trained by the Government of Ghana”* (Participant 8).

*“Of course, we have international standards to follow like the SDGs and we need skilled nurses to help us achieve such goals. So, if all the skilled nurses are leaving the country, leaving the less. It will affect our achievement of the SDGs”* (Participant 5).

#### 3.2.2. Challenges in Patient Care Quality and Satisfaction

The reduced compensation and increased workload could lead to the remaining nurses inadvertently expressing frustration towards patients, resulting in dissatisfaction with the care provided. Furthermore, there is concern about the potential decline in patient care standards due to the insufficient training of other cadres of nurses who may likely remain after most registered nurses have migrated. These staff may face challenges in implementing proper care plans, ultimately affecting patient care quality.

*“If there is more work and less pay, you will become frustrated. Some of us cannot hide our frustrations. We will also extend it to our clients. So, the clients will come and they are not satisfied with whatever activities or care that we have rendered to them rendering the whole health system useless because our optimum goal is to make our clients satisfied”* (Participant 2).

*“Nursing care plan has been one of the things that we want to implement but we are not able to do it because of the cadre of staff. The few diploma nurses with the volume of certificate nurses who they have, they don't learn it […] and for you to implement the nursing care plan, it needs much attention […] So, there is no way that they will be able to master it to be able to provide proper care, so low standard of care. That is what we are all wailing at, the low standard of care”* (Participant 4).

#### 3.2.3. Work Overload on the Remaining Nurses

Many participants emphasised that a significant consequence of nurses migrating abroad is the increased workload placed on the remaining nursing staff, adversely affecting their well-being. This augmented strain frequently impedes these nurses from dedicating adequate time to deliver the level of care that patients require. The overburdened staff face heightened stress levels, which can lead to burnout, decreased job satisfaction, and, ultimately, a further reduction in the quality of patient care. Additionally, the diminished workforce may struggle to maintain standard care protocols, potentially resulting in higher rates of medical errors and compromised patient outcomes. This scenario underscores the urgent need for strategies to support the remaining nursing staff, such as improving working conditions, ensuring adequate staffing levels, and providing mental health resources to sustain both nurse and patient well-being.

*“It is affecting us because the number of nurses is reducing and that is putting a strain on the remaining staff, eventually affecting the care we give to our patients”* (Participant 1).

Additionally, nurses' workload will include mentoring newly recruited nurses, as they represent the remaining pool of experienced nursing staff.

*“The burden on them would be like mentorship because that means that we have employed more like recruited new nurses, and they the few ones left have to begin to mentor them”* (Participant 16).

#### 3.2.4. Extended Patient Waiting Time

The participants observed that insufficient nursing staff attending to patients would result in longer wait times. This issue arises because the limited nursing workforce is required to complete ward rounds and other activities before they can address the needs of patients in critical areas, such as the Outpatient Departments (OPDs). Consequently, the time-sensitive demands of patients requiring immediate attention may not be met promptly, exacerbating patient dissatisfaction and potentially compromising patient outcomes. The cascading effects of these delays can strain the entire healthcare system, highlighting the critical need for adequate staffing to ensure efficient and effective patient care. Ensuring sufficient nursing personnel is essential for maintaining the flow of care delivery and upholding the standards of healthcare services.

*“[…] If we are many, at least some will be at the ward to carry out the rounds, and some will be at the OPD to take care of the patients. But if the numbers reduce, it will be like some few or one person. The person has to go forward rounds, finish, and then come to the OPD and attend to those waiting. So, the waiting time will increase automatically”* (Participant 12).

### 3.3. Mitigating Factors of the Brain Drain of Nurses

The third and final main category identified in this study was the mitigating factors of nurse brain drain, encompassing four subcategories: Opportunities for Career Advancement and Sponsorship, Improved Salary and Working Conditions, Provision of Attractive Health Infrastructure and Policies, and Provision of Health Insurance Coverage. Mitigating factors are crucial in addressing the brain drain of nurses. Critical insights into practical strategies for retention and recruitment within the nursing profession were uncovered through this research.

By analyzing these factors, this study illuminates actionable pathways for mitigating the adverse effects of nurse brain drain, ultimately contributing to enhancing healthcare delivery systems in Ghana. Implementing these strategies can foster a more stable and satisfied nursing workforce, which is essential for achieving sustainable improvements in healthcare quality and accessibility.

#### 3.3.1. Opportunity for Career Advancement and Sponsorships

Numerous participants emphasised the importance of providing career advancement opportunities for deserving nursing staff as a strategic measure to minimise nurse migration. They suggested that these opportunities should include full or partial sponsorship for the desired programs, thereby enhancing nursing personnel's professional development and retention. By investing in the career growth of nurses, healthcare institutions can foster a more committed and skilled workforce, reducing the propensity for migration and ensuring better continuity of care within the healthcare system.

*“If opportunities are given to deserving staff to pursue their career, they may stay”* (Participant 5).

*“Career development and advancement could help if only it will come with good incentives. As I stated, for instance, If I am going to develop myself […] and then all my fees are gone by 50% […] Why would I go? I would stay”* (Participant 9).

#### 3.3.2. Improved Salary and Working Conditions

Consistently, all participants proposed that a key strategy for addressing nursing migration is for the government to enhance nurses' salaries and working conditions. Improved compensation and working environments would strongly incentivise nurses to remain in the country. To mitigate the impact of the brain drain phenomenon, participants suggested that healthcare facilities should offer on-site accommodation to nurses. Additionally, participants recommended that the government introduce policies to standardise wages, ensuring fair and competitive compensation across the healthcare sector. These measures would improve nurse retention and promote a more stable and effective healthcare system.

*“The solutions are to improve nurses' salary and working conditions. If nurses' working conditions are improved, many people will not go away. When you are in your town or city, life will be better than going outside”* (Participant 8).

The participants also emphasised the importance of incentivising nurses working in remote areas. This is particularly significant due to the numerous challenges faced by these nurses, including inadequate access to basic necessities such as clean drinking water and electricity and poor road infrastructure. Providing additional incentives for nurses in these regions would acknowledge their unique hardships, encourage retention, and improve the quality of healthcare services in underserved areas.

*“Trained nurses don't want to go to some places to work mainly because the roads are terrible. If you have your family living in the city then you have to always travel all the way down”* (Participant 11).

#### 3.3.3. Provision of Attractive Health Infrastructure and Policies

Several participants suggested the government renovate aging hospital buildings to enhance their appeal. They also emphasised the importance of constructing new hospitals and facilities to ensure efficient patient management. It is crucial to ensure that basic items required for patient care are readily available in healthcare facilities. Furthermore, three of the sixteen participants proposed reinstating a commitment or bonding mechanism for nurses after completing their training. This measure could reduce the number of nurses who migrate abroad, helping retain skilled healthcare professionals within the country.

*“Government should make our hospitals attractive. The hospitals are old and there are no new buildings. At times it is the beauty of the hospital that attracts somebody to go and work there”* (Participant 3).

*“There should be policies in place that when you finish nursing school, you should serve the country for this number of years. After that, if you choose to leave, that's okay. But then it should be such that by the time those people decide to leave, you have people that have been trained also like mentorships. Then any point they exit, they have another set that can take care of the system”* (Participant 11).

#### 3.3.4. Provision of Health Insurance Coverage

Participants suggested that the government's implementation of a comprehensive health insurance policy for healthcare workers could significantly enhance nurse retention.

By providing robust health insurance coverage, the government would address a critical aspect of job satisfaction and security, thereby reducing the incentive for nurses to seek employment opportunities abroad. Comprehensive health insurance would improve the overall well-being of healthcare workers and demonstrate a tangible commitment to their welfare, which is essential for fostering a loyal and dedicated workforce. This policy could be a pivotal component in a broader strategy aimed at mitigating the adverse effects of brain drain within the nursing profession.

*“Another expectation is that if the government comes up with a policy that helps workers maybe health insurance policy […] that can also help them to stay […]”* (Participant 10).

## 4. Discussion

This study explored nurse managers' perspectives on the factors influencing and potential solutions for the brain drain of nurses in Northern Ghana. The multifaceted nature of this phenomenon indicates that migration is not driven by a single factor alone. Financial challenges, opportunities for professional growth, social considerations, workplace conditions, and political factors all play significant roles in nurses' decisions to migrate. The findings highlight the issue's complexity, underscoring the need for comprehensive and multifactorial strategies to effectively address the brain drain in the nursing sector [24].

Our research found that economic factors, such as low salaries and the desire to provide a better life for their families, are significant contributors to the nursing brain drain. These findings align with previous studies, which have similarly identified meagre salaries [3, 11, 25–27] and the desire to secure a better life for family [10, 28] as primary reasons for nurses leaving their home countries. Similarly, Lanati and Thiele [29] revealed nurses' migration is largely due to push factors such as economic challenges (low average salary) and pull factors including higher wages, improved quality of life, growing economy, and prestigious educational opportunities.

Moreover, this research underscores the critical role of working conditions in exacerbating nurse emigration, contributing significantly to brain drain. Previous studies consistently underscore the pivotal role of the work environment on nurses' decisions to seek opportunities abroad. For example, recent research elucidated how the scarcity of essential consumables and equipment is a compelling factor driving nurses to relocate to other nations—a finding congruent with existing literature [30, 31].

Improving workplace conditions and introducing effective financial incentives are crucial to minimising nurse migration issues due to economic factors and challenging work environments. This dual approach addresses nurses' financial needs and job satisfaction, fostering retention and strengthening healthcare systems.

Furthermore, the absence of opportunities for skill enhancement and career advancement, coupled with delays in promotions, has been identified as a key driver of nurse migrations. This finding reinforces the conclusions from comparable studies on the subject [10, 11, 32]. The government and the Ministry of Health should consider addressing this issue to tackle the challenges posed by brain drain in the country.

In our recent study, political factors, such as delays in recruiting graduate nurses and the recent policy of exporting nurses, emerged as significant determinants of nurse brain drain. This finding resonates with the research conducted by Poku et al. [10] which highlighted how political decisions impact nurse migration. Specifically, nursing graduates often face prolonged periods of unemployment, waiting over two years before securing a job due to bureaucratic delays. The recent practice of nurse exportation by the government makes nurses perceive an oversupply of trained professionals, further encouraging migration. To address this issue, policymakers should consider implementing reforms to streamline the recruitment process for nursing graduates, reduce waiting times for employment, and enhance transparency in government policies regarding nurse exports. Providing clear information on the rationale behind such decisions can help alleviate concerns and discourage unnecessary migration, ultimately mitigating nurse brain drain.

Our findings highlight that brain drain exacerbates work overload among remaining nurses, diminishing patient satisfaction. As nurses pursue better opportunities abroad, the patient-to-nurse ratio increases, necessitating the remaining staff to shoulder the workload. This finding resonates with similar studies by Yuksekdag [33]; Poku et al. [10]; Kadel and Bhandari [34]; and Rolle et al. [35], which have all documented the added burden on nursing personnel during periods of brain drain. Increased nurse workload is linked to reduced patient satisfaction and poorer outcomes [36, 37]. This correlation underscores the importance of advocating for humanistic care in nursing practice.

By fostering a culture of empathy and patient-centred care, we can strengthen the nurse-patient relationship, elevate the quality of nursing services, enhance patient satisfaction, and bolster professional recognition for nurses [38].

The present study revealed that the brain drain among nurses leads to newly trained nurses being introduced into the healthcare system with fewer experienced mentors available. Experienced nurses who leave take with them valuable knowledge and skills acquired over years of practice, leaving newly recruited nurses without the necessary expertise and guidance. Evidence indicates that patients' perceptions of hospital care are strongly associated with missed nursing care, directly related to inadequate professional nurse staffing [39–41]. For example, the study conducted by Karaca and Durna [42] revealed that patients were more satisfied with the care provided by experienced nurses, as the nursing care they received during hospitalisation was excellent. Therefore, the government could set up mentorship programs, supportive work environments, and opportunities for continuous training and skill development so that the remaining nurses can advance their knowledge and skills to provide quality care to patients.

Our research underscores that the continual migration of nurses poses a significant obstacle to attaining the Sustainable Development Goals (SDGs). This challenge is closely tied to the International Council of Nurses [43] assertion that nurses play a pivotal role in achieving the SDGs and that without adequate investment in the nursing profession, success in these global objectives is unattainable. The SDGs, established in September 2015 with the initiative of the World Health Organization (WHO), aim to foster measurable progress across social, economic, and environmental dimensions by 2030. Nurses are vital to this endeavour, serving as primary healthcare providers in all communities and settings. Their crucial role is highlighted by the fact that they outnumber doctors by nearly three to one [44]. However, an uneven distribution of nursing personnel weakens health systems, as Peters et al. [37] noted, ultimately hindering SDG targets' achievement. Addressing the challenges of nurse migration is crucial for the sustainability of healthcare systems and for realising the broader goals of global development outlined in the SDGs.

This study found that ongoing nurse brain drain increases patient waiting time. This may be explained by the reduced number of nurses in the healthcare system and hospitals when they migrate to foreign countries, especially in the absence of active recruitment efforts to replace those who have left. Waiting time indicates the quality of healthcare services [45, 46]. Prolonged waiting times significantly contribute to patient dissatisfaction [47, 48] and can have negative impacts on patient adherence to medication treatment regimens, clinical outcomes, the likelihood of patients returning to see their care providers, and the probability of patients recommending their care providers to others [49].

The current study underscores the pivotal role of government initiatives in creating an empowering work environment conducive to nurse retention. A predominant theme among participants was the desire for support in continuing their education through training and avenues for career advancement. They believed such support would enhance their personal development and professional standing and enable them to serve their communities better. This sentiment aligns with findings from research by Iwu [50] and Lartey et al. [51] which highlighted the effectiveness of providing opportunities for continuing education, career progression, and fostering a supportive workplace culture in stemming the nursing exodus. Furthermore, insights from Poudel et al.'s [52] study on nurses in preregistration programs in Nepal shed light on the motivations behind migration aspirations. The majority desired to migrate, with many citing access to educational opportunities abroad as a primary driver. However, they agreed they would stay in their home country if similar educational prospects were available locally. This underscores the importance of not only providing educational opportunities but also ensuring their accessibility to nurses, thereby fostering retention and mitigating the urge to migrate.

A significant finding of this study is that providing accommodation for nurses could be an effective strategy to mitigate brain drain. When implemented by healthcare organizations, this practical solution alleviates the financial burden of renting from external landlords and allows nurses to concentrate more fully on their professional responsibilities. These findings, in line with the research conducted by Adzei and Atinga [53], underscore the importance of housing as a crucial incentive for retaining healthcare personnel within a nation, providing healthcare administrators, policymakers, and researchers with actionable insights [54].

## 5. Conclusion

The study underscores numerous factors contributing to the emigration of Ghanaian nurses, which consequently leads to significant adverse effects on the healthcare system. These include heightened workload among remaining nurses [55], diminished patient satisfaction, lowered standards of care, a shortage of skilled professionals, impeded progress towards Sustainable Development Goals (SDGs), and prolonged patient waiting times. Effectively addressing this challenge necessitates a comprehensive approach. This involves implementing various potential solutions, such as offering career advancement pathways, enhancing salary and working conditions, bolstering health infrastructure, ensuring equitable pay, providing health insurance coverage, maintaining adequate supplies of consumables and equipment, offering accommodation support, and implementing bonding arrangements. It is crucial to address these multifaceted issues comprehensively to mitigate nurse brain drain and strengthen the resilience of Ghana's healthcare system.

### 5.1. Limitations of the Study

While this study shed light on the determinants and mitigating factors of the brain drain among Ghanaian nurses, it focused only on nurse managers in Northern Ghana, restricting the generalizability of the findings to a broader population of Ghanaian nurses. Also, there might be bias in selecting participants, especially if nurse managers who agreed to participate have distinct views or experiences compared to those who declined.

## Figures and Tables

**Table 1:** The sociodemographic profile of the participants in this study.

Participant	Age in years	Gender	Work experience	Qualification	Considering or intending to work abroad
Participant 1	58	Female	24	Bachelors	No

Participant 2	48	Female	25	Bachelors	No

Participant 3	56	Female	31	Bachelors	No

Participant 4	40	Female	16	Masters	Yes

Participant 5	35	Male	10	Bachelors	Yes

Participant 6	33	Male	6	Masters	Yes

Participant 7	41	Male	14	Bachelors	Yes

Participant 8	38	Male	13	Bachelors	Yes

Participant 9	36	Male	14	Masters	Yes

Participant 10	42	Female	13	Masters	Yes

Participant 11	36	Female	13	Masters	Yes

Participant 12	39	Female	14	Masters	Yes

Participant 13	39	Female	18	Masters	Yes

Participant 14	39	Male	14	PhD	Yes

Participant 15	40	Male	17	Bachelors	No

Participant 16	37	Female	10	Masters	Yes

Mean ± SD or total (%)	41.0 ± 7.1	Female: 9 (56.2%) Male: 7 (43.8%)	16.0 ± 6.3	Bachelors: 7 (43.8%) Masters: 8 (50.0%) PhD: 1 (6.2%)	Yes: 12 (75.0%) No: 4 (25.0%)

Source: field data (2023).

## Data Availability

The data supporting the conclusions of this study are available upon request from the corresponding author.
